# Patient-Centred Communication and Behavioural Guidance: An Exploratory Evaluation of the Trainer–Doctor Model in Dental Practice

**DOI:** 10.3390/healthcare14121759

**Published:** 2026-06-18

**Authors:** Lucian Josan, Elena Gabriela Strete, Alina Ormenișan, Ioana Cristina Talpos-Niculescu, Diana Marian, Andreea Salcudean, Ana Gabriela Seni, Iustin Olariu

**Affiliations:** 1 Private Medical Practice, 510125 Alba Iulia, Romania; lucian.josan@umfst.ro; 2 OMF Department, George Emil Palade University of Medicine, Pharmacy, Science and Technology (UMFST), 540142 Târgu Mureș, Romania; alina.ormenisan@umfst.ro; 3 Department of Psychiatry, George Emil Palade University of Medicine, Pharmacy, Science and Technology (UMFST), 540142 Târgu Mureș, Romania; elena.buicu@umfst.ro; 4 Clinic of Oro-Dental Diagnosis and Ergonomics, “Victor Babes” University of Medicine and Pharmacy, 300041 Timisoara, Romania; 5 Department of Dentistry, Faculty of Dentistry, “Vasile Goldiș” Western University of Arad, 94-96 Revolutiei Blvd., 310025 Arad, Romania; olariu.iustin@uvvg.ro; 6 Department of Ethics and Social Sciences, George Emil Palade University of Medicine, Pharmacy, Science and Technology (UMFST), 540142 Târgu Mureș, Romania; andreea.salcudean@umfst.ro; 7 Doctoral School of Faculty of Medicine, George Emil Palade University of Medicine, Pharmacy, Science and Technology (UMFST), 540142 Târgu Mureș, Romania; gabriela.seni@umfst.ro

**Keywords:** patient-centred care, health communication, patient engagement, behavioural medicine, dental education, questionnaire validation

## Abstract

Background/Objectives: The trainer–doctor model (TDM) is a participatory paradigm in which the physician acts as a mentor and educator. Effective health communication and patient engagement are key determinants of treatment adherence and health outcomes. Based on this conceptual framework, the present study aimed to assess preferences for the Trainer–Doctor Model among dental practitioners and patients, examine the influence of demographic variables, and provide a preliminary psychometric evaluation of the TDM questionnaire in accordance with the COSMIN (COnsensus-based Standards for the selection of health Measurement Instruments) criteria. Methods: A cross-sectional survey was conducted in Romania between May 2023 and April 2024. The study included dental practitioners recruited during scientific dental conferences and patients recruited from a private dental practice in Alba Iulia, Romania. Eligible participants were adults aged 18 years or older who provided written informed consent and completed the data protection requirements. Individuals younger than 18 years of age or those who did not provide complete informed consent were excluded. Participants completed a 12-item Likert-type questionnaire assessing preferences toward the Trainer–Doctor Model. Results: Both groups showed high TDM preference (practitioners: 43.93 ± 5.56; patients: 44.77 ± 4.84; *p* = 0.195); 71–76% of responses were high-preference (≥4). Cronbach’s α with reverse-scored items was 0.752/0.651. EFA (KMO = 0.740; Bartlett’s *p* < 0.001) identified a 3-factor structure, explaining 51.3% of the variance. Patients scored significantly higher on items A (*p* = 0.002), B (*p* = 0.022), and F (*p* = 0.005). Conclusions: Both groups demonstrate a strong, consistent preference for TDM across demographics. The preliminary psychometric evaluation indicates acceptable internal consistency and structural validity; however, further validation, including Delphi-based content validation and confirmatory factor analysis, is required.

## 1. Introduction

The doctor–patient relationship has undergone a significant transformation, evolving from a paternalistic model toward participatory and collaborative approaches such as shared decision-making and patient-centred care [1,2]. Within this context, the Trainer–Doctor Model (TDM) can be conceptually situated within broader frameworks of patient-centred care, shared decision-making, and health behaviour change models. While these paradigms emphasise communication, autonomy, and mutual agreement, the TDM introduces an additional, distinct dimension: the formative role of the physician, highlighting the clinician’s responsibility not only for diagnostic and therapeutic decision-making but also for guiding and shaping patient behaviour [3,4,5,6,7,8].

Although TDM overlaps with patient-centred communication, shared decision-making, health coaching, and lifestyle counselling, its distinctive feature is the explicit placement of the physician’s trainer/mentor role at the centre of the doctor–patient relationship. In patient-centred communication [9], the emphasis is primarily on understanding the patient’s perspective, building a therapeutic relationship, and supporting informed decisions [10]. In shared decision-making, the focus is on a collaborative choice between the clinician and the patient. In contrast, TDM adds a more explicit formative dimension, in which the physician not only communicates and collaborates, but also educates, guides, and reinforces health-related behaviours over time. This distinction is particularly relevant in preventive and chronic-care contexts, where adherence and sustained behavioural change are essential [11,12].

However, this model also presents important challenges. First, expanding the physician’s role toward continuous educational and behavioural guidance may increase workload and contribute to professional burden or burnout if not supported by appropriate systems, time allocation, and team-based care. Second, the quality of TDM implementation may vary with the clinician’s communication style, pedagogical skills, professional experience, and personal approach, as well as with the patient’s educational level, health literacy, motivation, and disease severity. Third, although physicians are trained to diagnose, treat, communicate, and work within clinical teams, they are not always formally trained as educators or behavioural coaches. Therefore, the practical use of TDM should not assume that all physicians are naturally prepared to teach effectively; rather, it requires structured communication training, educational strategies, and integration with existing patient-centred models.

In this context, effective health communication and patient engagement have become central components of high-quality healthcare delivery [3,4,5,6,13,14,15,16,17]. The TDM framework may offer a structured approach to understanding how physician-led behavioural guidance contributes to patient-centred care, treatment adherence, and preventive health behaviour, particularly in dentistry, where clinical outcomes frequently depend on patient understanding, cooperation, and sustained behavioural change [14,15,16,17].

The etymological origin of “doctor” as “teacher” underscores this dimension: the physician not only treats but educates, shaping patient conduct beyond the clinical encounter [1]. In the TDM framework, therapeutic decisions are jointly formulated, with the physician leveraging mentoring capacity to instil appropriate behaviours individualised for each patient [2].

Key characteristics of the TDM include: (1) it is a participatory model based on shared decision-making; (2) it acknowledges the transition from individual to patient status; (3) it prioritises patient well-being through behavioural guidance and health promotion; (4) it emphasises the physician’s educational and mentoring role as an applied dimension of existing communication, interpersonal, health promotion, and patient-care competencies; and (5) the relationship is built on mutual trust, holistic assessment, and rational therapeutic guidance [3,4,10]. Thus, within the TDM framework, the physician’s formative role should be understood not as a newly proposed competency, but as a practical integration of established competencies related to communication, patient education, health promotion, and maintenance of oral health.

Modern healthcare increasingly recognises the importance of patient-centred communication, particularly in dentistry, where compliance depends heavily on patient understanding and cooperation [14,15,16,17]. Digital platforms, online health communities, and artificial intelligence have introduced new dynamics [18,19,20]. The doctor–patient relationship has evolved from a paternalistic model toward participatory approaches such as shared decision-making, which emphasise patient autonomy, communication, and collaborative decision processes [21].

In this sense, TDM may be interpreted as a complementary framework that structures and measures the physician’s formative and behavioural guidance role within existing patient-centred approaches.

Previous studies have discussed the Trainer–Doctor Model in dental and oral health contexts, particularly regarding patients’ interest in this type of doctor–patient relationship and its association with self-efficacy. However, available evidence remains limited, and the model has not yet undergone comprehensive psychometric validation across diverse clinical settings. Therefore, the present study should not be interpreted as the first conceptual use of TDM, but rather as an exploratory application in dental practice and a preliminary psychometric evaluation of a newly developed TDM questionnaire.

To improve conceptual clarity, a schematic representation of the TDM framework and its relationship with existing patient-centred approaches is provided as Supplementary Figure S1.

Based on this conceptual framework, the present study aimed to assess preferences for the Trainer–Doctor Model among dental practitioners and patients, examine the influence of demographic variables, and provide a preliminary psychometric evaluation of the TDM questionnaire in accordance with the COSMIN (COnsensus-based Standards for the selection of health Measurement Instruments) criteria [22]. The COSMIN framework provides methodological standards for evaluating the quality of health-related measurement instruments, including their reliability, validity, and interpretability. Its use is particularly relevant when newly developed questionnaires are assessed in exploratory validation studies [22].

## 2. Materials and Methods

### 2.1. Study Design and Population

A cross-sectional survey was conducted in Romania between May 2023 and April 2024. The study population comprised two main groups: dental practitioners and patients. Dental practitioners were recruited during scientific dental conferences held in Romania, whereas patients were recruited from the Private Medical Practice Dr Josan Lucian, Alba Iulia, Romania. Thus, the two groups were recruited from different settings: a professional scientific setting for dental practitioners and a clinical private practice setting for patients. The study was approved by the Ethics Committee of the George Emil Palade University of Medicine, Pharmacy, Science, and Technology of Târgu Mureș, Romania (Decision No. 2311, 26 April 2023), and was conducted in accordance with the Declaration of Helsinki. Written informed consent was obtained from all participants.

### 2.2. Inclusion and Exclusion Criteria

Participants were eligible for inclusion if they were adults aged 18 years or older, provided written informed consent, and completed the General Data Protection Regulation (GDPR) consent requirements. Dental practitioners were included if they were actively involved in dental practice, while patients were included if they attended the participating private dental practice during the study period and agreed to complete the questionnaire. Individuals younger than 18 years of age, those who did not provide complete informed consent, or those who did not complete the questionnaire were excluded from the study.

### 2.3. Questionnaire Instrument

The TDM questionnaire was generated by the authors as a newly developed instrument intended to operationalise the physician’s formative, educational, and behavioural guidance role within the Trainer–Doctor Model. Item generation was informed by the conceptual framework of the TDM and by literature on patient-centred communication, shared decision-making, health coaching, patient education, and behavioural guidance. The instrument had not undergone formal qualitative item generation or full psychometric validation before the present study; therefore, the current analysis should be interpreted as an exploratory preliminary psychometric evaluation.

The questionnaire comprised 12 items, labelled A–L, scored on a 6-point Likert scale ranging from 0 = “not at all” to 5 = “to a very great extent”. The items were organised into complementary conceptual pairs: A/G, B/H, C/I, D/J, E/K, and F/L. This pairing was intended to capture related dimensions of the TDM construct from different perspectives, including physician guidance, patient reliance on medical expertise, perceived security, interest in professional competence, and acceptance of therapeutic recommendations.

Items G and I were formulated in a direction opposite to the main TDM construct and were therefore reverse-scored using the transformation 5 − x. After reverse scoring, higher scores consistently indicated a stronger preference for the Trainer–Doctor Model. A total TDM score was calculated by summing the item scores after reverse scoring where applicable. The full TDM questionnaire, including all 12 items and the response scale, is provided as Supplementary File S2 to improve transparency and allow readers to assess the content of the instrument.

### 2.4. Variables

The variables collected in this study included participant group, demographic characteristics, and TDM questionnaire outcomes. The participant group was defined as a dental practitioner or a patient. Demographic variables included age, sex, residential environment (urban or rural), and educational level (pre-university, university, or postgraduate). TDM questionnaire outcomes consisted of individual responses to the 12 questionnaire items, labelled A–L, each scored on a 6-point Likert scale ranging from 0 = “not at all” to 5 = “to a very great extent”. Items G and I were reverse-scored for psychometric analyses. A total TDM score was calculated by summing the item scores after reverse scoring where applicable. For descriptive and comparative analyses, individual item responses were also dichotomised into high preference (scores ≥ 4) and low preference (scores < 4). This threshold was selected to reflect a clinically meaningful level of agreement and to facilitate interpretation of response patterns.

Derived variables used in subgroup analyses included age group, sex, and residential environment for both groups, while educational level was analysed as a subgroup variable only among patients. Psychometric variables included internal consistency indices, inter-item correlations, factor loadings, communalities, and factor structure obtained through exploratory factor analysis.

### 2.5. Statistical Analysis

Statistical analysis was performed using Python 3.12 (Python Software Foundation, Wilmington, DE, USA; https://www.python.org),with SciPy 1.14, pandas 2.2, and factor_analyzer 0.5.0. Normality was assessed using the Shapiro–Wilk test. As the data showed deviations from normality, non-parametric tests were applied. Group comparisons between dental practitioners and patients were performed using the Mann–Whitney U test, while comparisons across more than two subgroups were conducted using the Kruskal–Wallis H test. Categorical variables were analysed using Chi-square or Fisher’s exact tests, as appropriate. Associations between questionnaire items were assessed using Spearman’s rank correlation coefficients. Effect sizes were estimated using Cohen’s d, and statistical significance was set at *p* < 0.05.

For the psychometric evaluation of the TDM questionnaire, internal consistency was assessed using Cronbach’s alpha, calculated before and after reverse scoring items G and I. Structural validity was explored using exploratory factor analysis with principal axis factoring and Varimax rotation. Sampling adequacy was evaluated using the Kaiser–Meyer–Olkin measure, and the suitability of the correlation matrix for factor analysis was assessed using Bartlett’s test of sphericity. The number of factors was examined using parallel analysis and Kaiser’s criterion, with final factor retention guided by both statistical results and theoretical interpretability. Construct validity was evaluated using Spearman correlations between theoretically paired items and known-groups comparisons between dental practitioners and patients.

### 2.6. Psychometric Evaluation Framework

The psychometric evaluation of the TDM questionnaire was conducted as an exploratory preliminary validation process, guided by the COSMIN Risk of Bias checklist [22]. The assessment focused on internal consistency, structural validity, construct validity, and known-groups validity. Content validity was not formally assessed in the present study; therefore, the questionnaire should not yet be considered fully validated. A structured content validation process using a modified Delphi method is planned for future research, involving an expert panel and predefined thresholds for item-level and scale-level content validity indices. Test–retest reliability, measurement error, confirmatory factor analysis, and cross-cultural validation should also be addressed in subsequent studies.

## 3. Results

### 3.1. Baseline Characteristics of Study Participants

The baseline characteristics of the study participants are presented in Table 1. The study included 107 dental practitioners and 157 patients. Practitioners had a mean age of 33.43 ± 8.60 years, with an observed age range of 23–69 years, while patients had a mean age of 39.14 ± 14.42 years, with an observed age range of 18–74 years. Both groups were predominantly female and from urban areas.

### 3.2. TDM Item Scores

**Figure 1:**
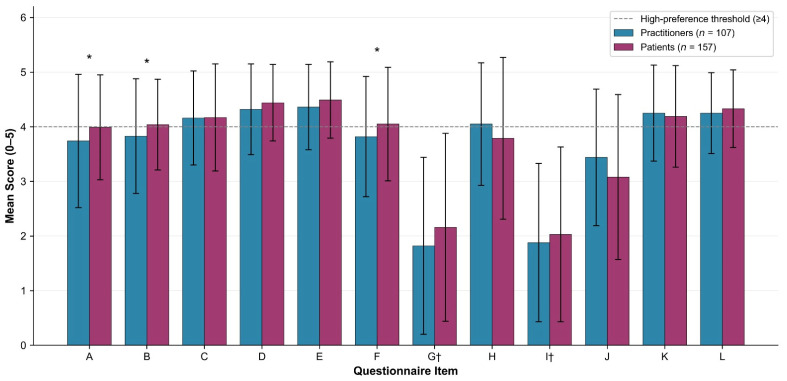
Mean TDM item scores ± SD for dental practitioners and patients. TDM = Trainer–Doctor Model; SD = standard deviation; ^†^ reverse-scored item; * *p* < 0.05.

The mean TDM item scores for practitioners and patients are illustrated in Figure 1. Detailed descriptive statistics for each item and between-group comparisons are presented in Table 2. Significant differences between groups were observed for items A, B, and F (*p* < 0.05).

**Figure 2:**
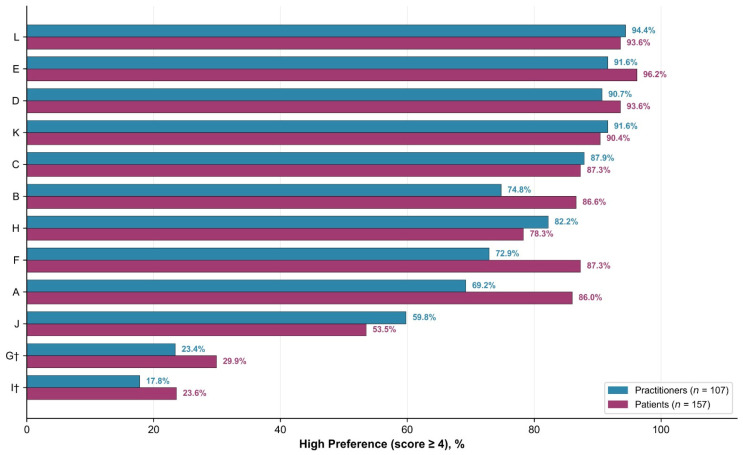
Proportion of high preference responses (score ≥ 4) by item, ^†^ reverse-scored item.

The proportion of high-preference responses is shown in Figure 2. Detailed item-level percentages are provided in Table 2; therefore, only the main between-group pattern is highlighted here. Patients showed significantly higher endorsement for items A, B, and F, while the remaining items did not differ significantly between groups.

### 3.3. Response Distribution Profiles

**Figure 3:**
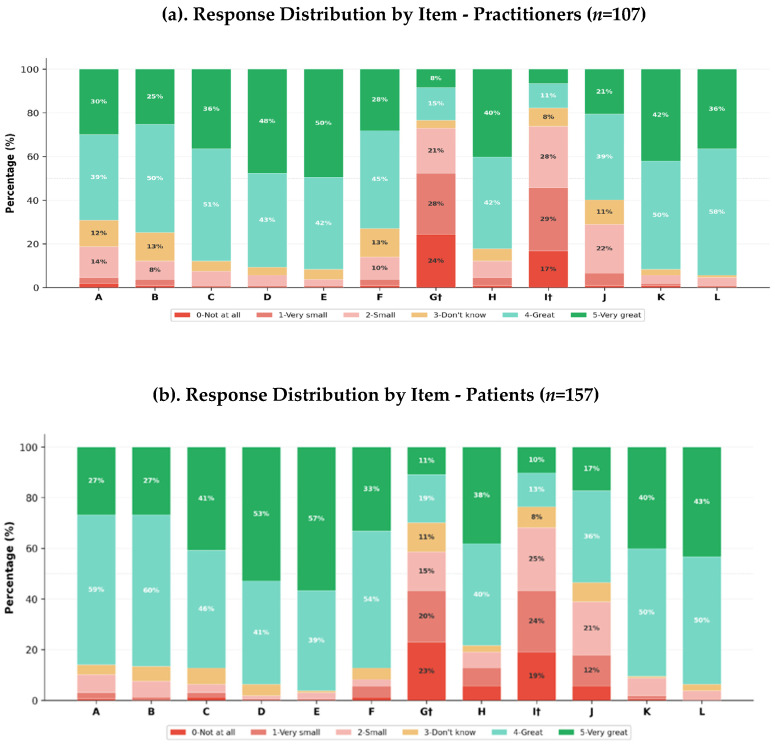
Response distribution (%) by item. (**a**) Practitioners (*n* = 107); (**b**) Patients (*n* = 157). ^†^ reverse-scored item.

The distribution of responses across all items is presented in Figure 3, separately for practitioners and patients, illustrating overall response patterns within each group.

### 3.4. Total TDM Score by Demographic Subgroups

**Figure 4:**
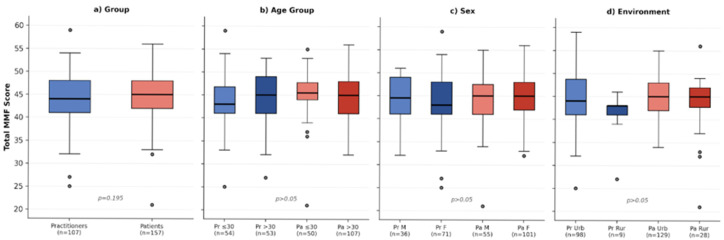
Total TDM score by (**a**) group, (**b**) age, (**c**) sex, (**d**) environment. All *p* > 0.05. Blue boxes: practitioners (Pr); red boxes: patients (Pa). Boxes show the interquartile range with the median line; whiskers extend to 1.5 × the interquartile range; dots indicate outliers.

Total TDM scores across demographic subgroups are illustrated in Figure 4, with detailed comparisons provided in Table 3. No statistically significant differences were observed according to participant group, age group, sex, residential environment, or patient educational level.

### 3.5. TDM Preference Profile

**Figure 5:**
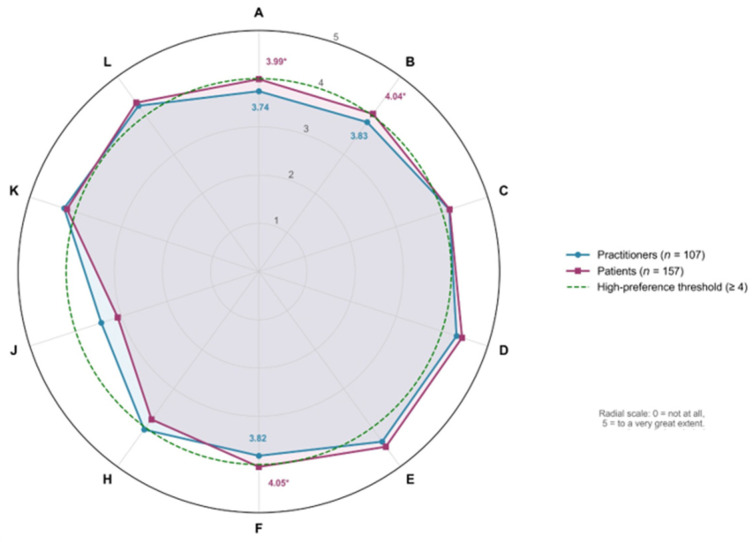
Radar plot of mean TDM item scores (non-reverse items). Dashed green circle = high-preference threshold (≥4). * *p* < 0.05.

The overall TDM preference profile is visualised in Figure 5, showing mean item scores relative to the high-preference threshold.

### 3.6. Internal Consistency and Inter-Item Correlations

**Figure 6:**
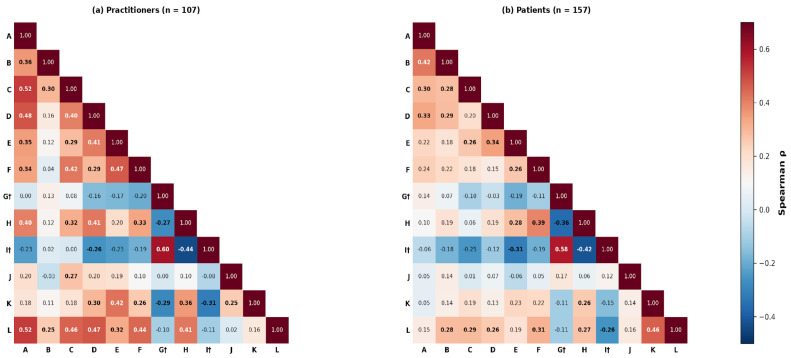
Spearman correlation matrices for dental practitioners (**a**) and patients (**b**). TDM = Trainer–Doctor Model. ^†^ reverse-scored item.

Inter-item correlations and internal consistency results are presented in Figure 6 and Table 4. Internal consistency improved after reverse-coding items G and I.

### 3.7. Patient Subgroup by Educational Level

**Figure 7:**
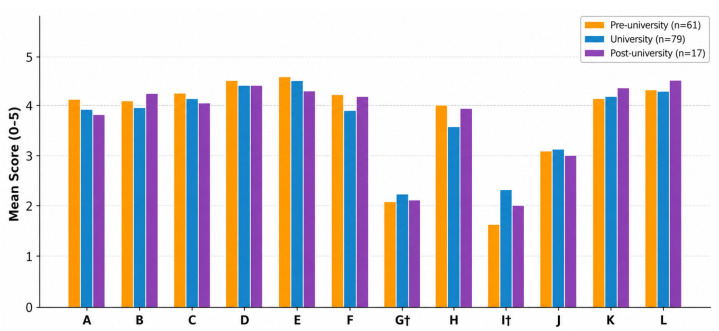
TDM scores by patient educational level. Kruskal–Wallis H = 0.12, *p* = 0.942. ^†^ reverse-scored item.

Differences in TDM scores by patient educational level are shown in Figure 7, with no statistically significant differences identified.

### 3.8. Structural Validity: Exploratory Factor Analysis

**Figure 8:**
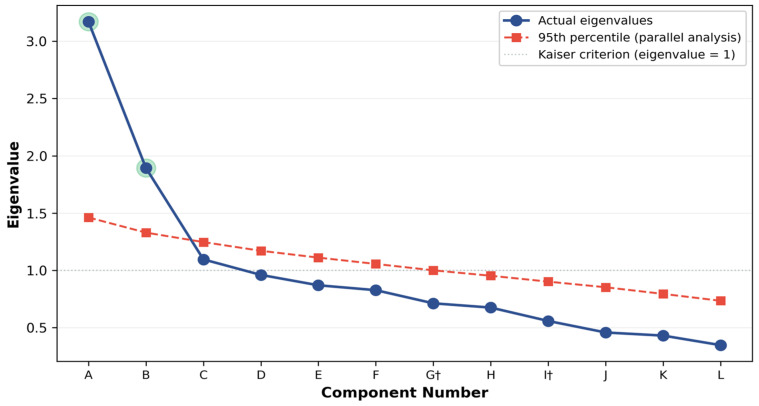
Scree plot with parallel analysis.. Blue line with circular markers: observed eigenvalues; dashed red line: 95th percentile of eigenvalues from random data (parallel analysis, Horn’s method); dotted grey line: Kaiser criterion (eigenvalue = 1); green circles: factors indicated by parallel analysis. ^†^ reverse-scored item.

**Figure 9:**
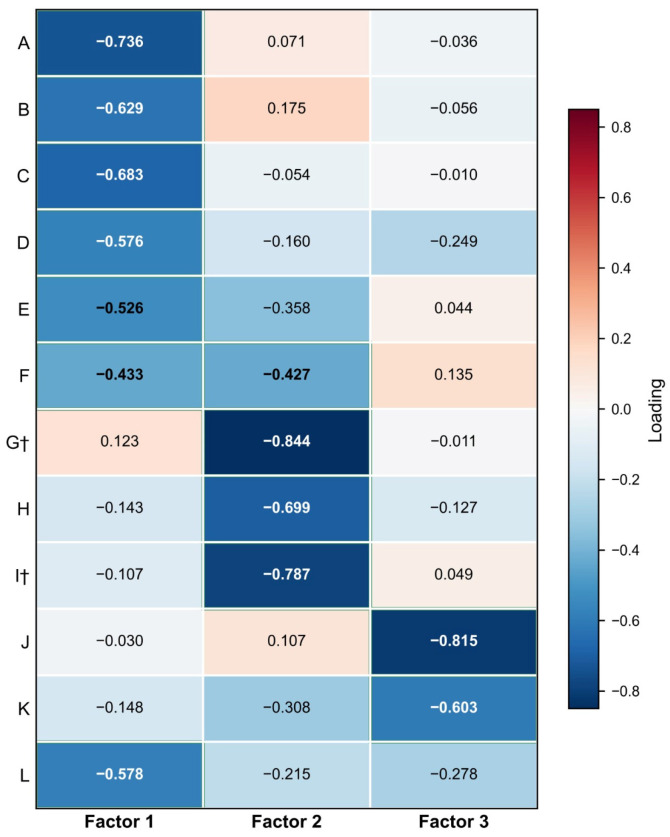
Factor loading matrix (3-factor Varimax, *n* = 264). Green borders: |loading| ≥ 0.40. ^†^ reverse-scored item.

EFA was performed on the combined sample after reverse-scoring items G and I. Sampling adequacy was acceptable (KMO = 0.740), and Bartlett’s test of sphericity was significant (*p* < 0.001). The scree plot and parallel analysis results are presented in Figure 8; parallel analysis suggested a two-factor solution, whereas Kaiser’s criterion (eigenvalue > 1) supported three factors. The three-factor solution was conceptually interpretable and included Physician’s Formative Role, Medical Necessity, and Health Security. Detailed rotated factor loadings and communalities are presented in Table 5 and visually summarised in Figure 9.

### 3.9. COSMIN Psychometric Summary

The psychometric properties of the TDM questionnaire, assessed according to COSMIN criteria, are summarised in Table 6.

## 4. Discussion

The present study provides preliminary quantitative evidence regarding the acceptance of the Trainer–Doctor Model among both dental practitioners and patients, supporting the relevance of an educational and participatory approach within the contemporary doctor–patient relationship. The high proportion of favourable responses should be interpreted with caution, as it may reflect a combination of genuine preferences and potential social desirability bias inherent in self-reported measures that address normative aspects of the physician–patient relationship. The TDM construct should be interpreted as an extension of the shared decision-making paradigm, integrating elements of health coaching and patient education. Unlike traditional patient-centred models that emphasise shared responsibility, the TDM framework emphasises a structured professional guidance component, in which the physician maintains an active role in supporting and shaping patient behaviour, particularly in preventive contexts such as dentistry. Most importantly, this study attempts to operationalise a conceptual model that has previously been discussed in relation to the doctor–patient relationship but has not yet been extensively evaluated using a dedicated psychometric instrument [2]. While the principles of shared decision-making and patient-centred care are well established in the literature, the TDM framework places a distinct emphasis on the physician’s formative and mentoring role, particularly in influencing patient behaviour, adherence, and long-term health-related behaviour. In this respect, the model extends beyond the traditional communication paradigm by integrating educational responsibility as an applied dimension of established communication, health promotion, and patient care competencies.

The three-factor structure identified through exploratory factor analysis—Physician’s Formative Role, Medical Necessity, and Health Security—appears to be conceptually coherent and potentially clinically meaningful. The first factor captures the central TDM construct, namely the physician’s role as educator and behavioural guide. The second factor reflects the recognition of professional medical expertise and the limits of self-management. In contrast, the third factor highlights the psychological dimension of illness-related vulnerability and the need for reassurance through professional competence. Cumulative variance (51.3%) is acceptable; KMO (0.740) confirms adequate sampling [23]. Patients scored significantly higher on items A (*p* = 0.002), B (*p* = 0.022), and F (*p* = 0.005), suggesting idealisation of the physician’s formative role. This expectation gap aligns with Birkeland et al. [8]. Cronbach’s α improvement with reverse scoring (0.572 → 0.752; 0.347 → 0.651) confirms G, and I function as intended. Patient α (0.651) remains acceptable for a new instrument [24]. No significant demographic differences in total TDM scores (Cohen’s d < 0.3), indicating broad acceptance. These findings are particularly relevant in dentistry, where treatment success frequently depends on patient cooperation, oral hygiene compliance, and sustained behavioural modification.

Previous research has demonstrated that communication significantly influences patients’ understanding, adherence, and treatment outcomes, while psychosocial factors such as dental anxiety may further affect patients’ behaviour and perceptions of care [25,26]. Beyond individual behavioural and psychological factors, access to dental care represents an important determinant of oral health behaviour, particularly in rural communities, where structural and socioeconomic barriers may influence both patient engagement and treatment adherence [27]. These findings further support the importance of behavioural guidance within the doctor–patient relationship. Health-related behaviours are key determinants of oral health outcomes, with evidence indicating that dietary habits and lifestyle factors are closely associated with oral health status and overall development, particularly when established early in life [28]. Moreover, studies on younger populations have shown that oral hygiene practices and dietary behaviours are closely interconnected, reinforcing the importance of early behavioural education in shaping long-term oral health outcomes [29]. These findings are consistent with previous research demonstrating that knowledge, attitudes, and preventive practices are closely interconnected, emphasising the critical role of education in shaping health-related behaviours and risk perception [30].

An important finding is the significantly higher scores observed among patients for items related to the physician’s formative influence (items A, B, and F). This may suggest that patients place greater value on the clinician’s educational and guiding role than practitioners themselves perceive. Such a discrepancy has important implications for dental education and professional development, underscoring the need to strengthen communication and interpersonal skills, patient education, health promotion, and counselling-based approaches in undergraduate and postgraduate curricula. These areas are consistent with established competency domains in dental education, particularly communication and interpersonal skills, health promotion, and patient care, rather than representing a separate, newly proposed competency [4,5]. From a psychometric perspective, the present study offers a preliminary validation of the TDM questionnaire. The acceptable internal consistency after reverse scoring and the coherent factorial structure support the instrument’s initial usability. However, these results should be interpreted as exploratory. The absence of formal content validation and confirmatory factor analysis limits the strength of the psychometric conclusions [22].

Beyond its exploratory nature, this study contributes to the ongoing refinement of patient-centred care by proposing a measurable framework that explicitly captures the physician’s formative role. The Trainer–Doctor Model should not be interpreted as a replacement for established approaches such as patient-centred care, shared decision-making, or health coaching. Rather, it may be used in conjunction with these models by adding a more explicit emphasis on the clinician’s role in educational and behavioural guidance. While shared decision-making focuses on collaboration, patient autonomy, and mutual agreement, TDM highlights the physician’s responsibility to guide, educate, and reinforce health-related behaviours over time. This complementary perspective may be particularly relevant in dentistry, where long-term outcomes frequently depend on adherence to preventive recommendations, oral hygiene practices, and sustained behavioural change.

### 4.1. Limitations

This study has several limitations that should be considered when interpreting the findings. First, the use of convenience sampling may have introduced selection bias. Dental practitioners were recruited during scientific dental conferences, whereas patients were recruited from a private clinical dental practice. Therefore, conference-attending practitioners may have been more professionally engaged or more interested in communication and behavioural guidance, whereas patients attending a private dental practice may differ from the general patient population in health literacy, motivation, socioeconomic background, or expectations regarding the doctor–patient relationship. Second, recruiting patients from a single private practice limits the representativeness and generalizability of the findings. In addition, the predominance of participants from urban areas further restricts the applicability of the results to broader or more diverse populations, including rural and public healthcare settings. Third, the cross-sectional design does not allow the assessment of temporal stability, causal relationships, or instrument responsiveness. Test–retest reliability and measurement error could not be evaluated in the present study and should be addressed in future longitudinal research. Fourth, the potential influence of social desirability bias should be acknowledged, as some questionnaire items may have encouraged responses aligned with perceived expectations regarding the physician’s role. Fifth, the practical implementation of the TDM framework may present additional challenges. Although the model emphasises the physician’s educational and behavioural guidance role, this may increase professional demands and contribute to workload or burnout if not supported by adequate time, institutional resources, and team-based care. Moreover, implementation quality may vary with the clinician’s communication style, pedagogical abilities, professional experience, and personal approach, as well as patient-related factors such as educational background, health literacy, motivation, and disease severity. Finally, physicians are not necessarily formally trained as educators or behavioural coaches; therefore, gaps in teaching expertise may limit the consistent application of the TDM in clinical practice.

Taken together, these limitations indicate that the findings should be interpreted as preliminary. Future multicentre studies should include more diverse recruitment settings and populations, as well as comprehensive psychometric validation procedures, including Delphi-based content validation, confirmatory factor analysis, test–retest reliability, measurement error assessment, and cross-cultural validation.

### 4.2. Future Directions

Future research should focus on the comprehensive validation and broader implementation of the TDM questionnaire. A key priority is assessing content validity through a structured modified Delphi process involving 20–25 experts, with predefined thresholds (I-CVI ≥ 0.78 and S-CVI/Ave ≥ 0.90) to ensure adequate item relevance and clarity [31]. Additionally, confirmatory factor analysis (CFA) should be conducted with a larger, more diverse sample (*n* ≥ 300) to verify the factorial structure identified in the present exploratory analysis. Longitudinal studies are also needed to evaluate test–retest reliability over a two-week interval, using intraclass correlation coefficients (ICC), as well as to assess measurement error and responsiveness [32]. Further research should include cross-cultural adaptation and validation of the TDM questionnaire across diverse linguistic and healthcare contexts to enhance its external validity and applicability across populations. From a practical perspective, integrating the Trainer–Doctor Model into dental education and continuous professional development programmes represents a promising direction. Future studies may explore the impact of TDM -based training on patient adherence, communication quality, and clinical outcomes [33].

## 5. Conclusions

The present study suggests that both dental practitioners and patients are generally receptive to the Trainer–Doctor Model, indicating that the physician’s educational and behavioural guidance role remains relevant within contemporary dental practice. Although overall TDM scores were similar between the two groups, higher patient endorsement of selected items related to the physician’s formative influence suggests that patients may expect clinicians to play a more active role in guiding, educating, and reinforcing health-related behaviours than practitioners themselves perceive.

These findings support the interpretation of TDM as a complementary framework to patient-centred care, shared decision-making, and health coaching, rather than as a replacement for these established models. Its potential value lies in making the clinician’s formative role more explicit and measurable, particularly in preventive and adherence-dependent areas of dentistry. At the same time, the absence of clear demographic associations suggests that the perceived relevance of this role may extend across participant categories, although this should be confirmed in broader, more diverse samples.

From a psychometric perspective, the preliminary findings suggest that the TDM questionnaire may serve as a useful starting point for assessing perceptions of physician-led behavioural guidance. However, the instrument remains exploratory and should not yet be considered fully validated. Future multicentre studies should combine comprehensive psychometric validation, including Delphi-based content validation and confirmatory factor analysis, with evaluation of the practical feasibility of TDM implementation, including clinician training needs, variability in application, and potential workload-related barriers.

## Figures and Tables

**Table 1:** Demographic characteristics of study participants.

Characteristic	Practitioners (*n* = 107)	Patients (*n* = 157)
Age, mean ± SD (range), years	33.43 ± 8.60 (23–69)	39.14 ± 14.42 (18–74)
Age ≤ 30/>30 years, *n* (%)	54 (50.5%)/53 (49.5%)	50 (31.8%)/107 (68.2%)
Female/Male, *n* (%)	71 (66.4%)/36 (33.6%)	101 (64.7%)/55 (35.3%)
Urban/Rural, *n* (%)	98 (91.6%)/9 (8.4%)	129 (82.2%)/28 (17.8%)
Education: Pre/Uni/Post, *n* (%)	1 (0.9%)/70 (65.4%)/36 (33.6%)	61 (38.9%)/79 (50.3%)/17 (10.8%)

SD = standard deviation; Pre = pre-university education; Uni = university education; Post = postgraduate education.

**Table 2:** Per-item descriptive statistics and between-group comparisons.

Item	Description	Pract. M ± SD	Pat. M ± SD	Pr H%	Pa H%	χ^2^	*p*
A	Health improvement requires a physician	3.74 ± 1.22	3.99 ± 0.96	69.2	86.0	9.93	0.002 *
B	Physician improves conduct	3.83 ± 1.05	4.04 ± 0.83	74.8	86.6	5.24	0.022 *
C	Physician helps fight illness	4.16 ± 0.86	4.17 ± 0.98	87.9	87.3	0.00	1.000
D	Health = security need	4.32 ± 0.83	4.44 ± 0.70	90.7	93.6	0.44	0.509
E	Competence guides choice	4.36 ± 0.78	4.49 ± 0.70	91.6	96.2	1.72	0.190
F	Accepts recommendations	3.82 ± 1.10	4.05 ± 1.04	72.9	87.3	7.76	0.005 *
G ^†^	Non-medical can heal	1.82 ± 1.62	2.16 ± 1.72	23.4	29.9	1.07	0.300
H	Needs a physician for therapy	4.05 ± 1.12	3.79 ± 1.48	82.2	78.3	0.38	0.535
I ^†^	Can self-heal	1.88 ± 1.45	2.03 ± 1.60	17.8	23.6	0.96	0.327
J	Illness induces insecurity	3.44 ± 1.25	3.08 ± 1.51	59.8	53.5	0.79	0.375
K	Interest in training level	4.25 ± 0.88	4.19 ± 0.93	91.6	90.4	0.01	0.921
L	Follows therapeutic conduct	4.25 ± 0.74	4.33 ± 0.71	94.4	93.6	0.00	1.000

Pract. = dental practitioners; Pat. = patients; M = mean; SD = standard deviation; H% = high-preference responses, defined as scores ≥ 4; ^†^ reverse-scored item; * *p* < 0.05.

**Table 3:** Total TDM score comparisons by demographic subgroups.

Comparison	Group 1	*n*	Mean ± SD	Group 2	*n*	Mean ± SD	*p*	d
Overall	Practitioners	107	43.93 ± 5.56	Patients	157	44.77 ± 4.84	0.195	−0.16
Pract. Age	≤30	54	43.67 ± 5.54	>30	53	44.19 ± 5.62	0.295	−0.09
Pat. Age	≤30	50	45.06 ± 5.07	>30	107	44.64 ± 4.73	0.371	0.09
Pract. Sex	M	36	44.06 ± 5.66	F	71	43.86 ± 5.55	0.937	0.04
Pat. Sex	M	55	44.38 ± 5.04	F	101	45.04 ± 4.72	0.718	−0.14
Pract. Milieu	Urban	98	44.19 ± 5.34	Rural	9	41.00 ± 7.78	0.142	0.58
Pat. Milieu	Urban	129	44.98 ± 4.65	Rural	28	43.82 ± 5.58	0.647	0.24
Pat. Edu	Pre	61	45.00 ± 4.43	Uni + Post	96	44.62 ± 5.09	0.732	0.08

Mann–Whitney U. Cohen’s d: small ≈ 0.2, medium ≈ 0.5, large ≈ 0.8.

**Table 4:** Internal consistency and paired-item Spearman correlations.

Measure/Item Pair	Practitioners	Patients
Cronbach’s α (raw)	0.572	0.347
Cronbach’s α (G, I reversed)	0.752	0.651
A vs. G	0.004 (*p* = 0.969)	0.140 (*p* = 0.081)
B vs. H	0.117 (*p* = 0.230)	0.188 (*p* = 0.019 *)
C vs. I	0.003 (*p* = 0.978)	−0.250 (*p* = 0.002 *)
D vs. J	0.204 (*p* = 0.035 *)	0.069 (*p* = 0.389)
E vs. K	0.425 (*p* < 0.001 *)	0.225 (*p* = 0.005 *)
F vs. L	0.444 (*p* < 0.001 *)	0.307 (*p* < 0.001 *)

* *p* < 0.05.

**Table 5:** Exploratory factor analysis: Varimax-rotated loadings (*n* = 264).

Item	Description	F1: Formative	F2: Necessity	F3: Security	h^2^
A	Physician role	−0.736	0.071	−0.036	0.548
B	Improving conduct	−0.629	0.175	−0.056	0.429
C	Fighting illness	−0.683	−0.054	−0.010	0.470
D	Security need	−0.576	−0.160	−0.249	0.419
E	Competence	−0.526	−0.358	0.044	0.406
F	Recommendations	−0.433	−0.427	0.135	0.388
G ^†^	Non-medical (rev.)	0.123	−0.844	−0.011	0.728
H	Need physician	−0.143	−0.699	−0.127	0.525
I ^†^	Self-healing (rev.)	−0.107	−0.787	0.049	0.633
J	Insecurity	−0.030	0.107	−0.815	0.676
K	Training interest	−0.148	−0.308	−0.603	0.481
L	Compliance	−0.578	−0.215	−0.278	0.457
SS loadings		2.604	2.346	1.210	
% Variance		21.7%	19.5%	10.1%	51.3%

F1 = Factor 1; F2 = Factor 2; F3 = Factor 3; h^2^ = communality; ^†^ reverse-scored item.

**Table 6:** COSMIN Risk of Bias assessment of the TDM questionnaire.

Property (COSMIN v3.0)	Rating	Evidence
Content validity	Inadequate	Author-designed; no Delphi. Planned.
Structural validity	Adequate	EFA: KMO = 0.74, 3 factors, 51.3%.
Internal consistency	Adequate	α = 0.752/0.651, G + I reversed.
Construct validity	Adequate	E/K ρ = 0.43, F/L ρ = 0.44, *p* < 0.001.
Known-groups	Adequate	Items A, B, F: *p* < 0.05.
Cross-cultural	Not assessed	Single-language (Romanian).
Test–retest	Not assessed	Cross-sectional. Planned.
Measurement error	Not assessed	SEM/SDC planned.
Criterion validity	N/A	No gold standard.
Responsiveness	N/A	Cross-sectional.

COSMIN = COnsensus-based Standards for the selection of health Measurement Instruments; TDM = Trainer–Doctor Model; EFA = exploratory factor analysis; KMO = Kaiser–Meyer–Olkin; SEM = standard error of measurement; SDC = smallest detectable change; N/A = not applicable.

## Data Availability

The data presented in this study are available on reasonable request from the corresponding authors: ioana.talpos-niculescu@umft.ro; marian.diana@uvvg.ro.

## Supplementary Materials

The following supporting information can be downloaded at: https://www.mdpi.com/article/10.3390/healthcare14121759/s1, Supplementary Figure S1: Conceptual representation of the Trainer–Doctor Model (TDM); Supplementary File S2: Trainer–Doctor Model Questionnaire.
