# Supplementary material for: Patient-Centred Communication and Behavioural Guidance: An Exploratory Evaluation of the Trainer–Doctor Model in Dental Practice
Source: Healthcare (Basel). 2026 Jun 18;14(12):1759. doi: 10.3390/healthcare14121759 (PMC13300019; doi:10.3390/healthcare14121759)
Supplement: Supplementary file 1 [file healthcare-14-01759-s001.zip › Supplementary File S2. Trainer–Doctor Model Questionnaire.pdf]

Supplementary File S2. Trainer–Doctor Model Questionnaire

The following questionnaire was used to assess participants' preferences regarding the Trainer–Doctor Model (TDM), with particular focus on the physician's formative, educational, and behavioural guidance role within the doctor–patient relationship.

Participants were asked to indicate the extent to which they agreed with each statement using the response scale below.

Response scale

0 = Not at all
1 = To a very small extent
2 = To a small extent
3 = I do not know / I cannot assess
4 = To a great extent
5 = To a very great extent

Questionnaire items

A. I believe that improving my health status as a patient can only be achieved with the help of a physician.
B. The physician can improve my conduct as a patient.
C. The physician helps me fight the disease/condition from which I suffer.
D. Optimal health represents a need for security.
E. The competence of my attending physician is a criterion in choosing them.
F. I readily accept the recommendations of my attending physician.
G. People without medical training can help me heal certain conditions.
H. A patient needs a physician in order to establish optimal therapeutic conduct.
I. I can heal without the help of specialised medical care.
J. Suffering from a certain condition induces a feeling of insecurity in me.
K. I am interested in the professional training level of my attending physician.
L. I understand that I should strictly follow the therapeutic conduct recommended by the physician.

Scoring note

Items are scored from 0 to 5, with higher scores indicating stronger agreement with each statement. Items G and I are reverse-scored because they are formulated in the opposite direction to the main Trainer–Doctor Model construct. After reverse scoring, higher total scores indicate a stronger preference for the Trainer–Doctor Model.
